# Characterization, developmental expression and evolutionary features of the huntingtin gene in the amphioxus *Branchiostoma floridae*

**DOI:** 10.1186/1471-213X-7-127

**Published:** 2007-11-15

**Authors:** Simona Candiani, Mario Pestarino, Elena Cattaneo, Marzia Tartari

**Affiliations:** 1 Department of Biology, University of Genoa, viale Benedetto XV 5, 16132, Genoa, Italy; 2 Centre for Stem Cell Research and Department of Pharmacological Sciences, University of Milan, Via Balzaretti 9, 20133, Milan, Italy

## Abstract

**Background:**

Huntington's disease is an inherited neurodegenerative disorder that is caused by the expansion of an N-terminal polyQ stretch in the huntingtin protein. In order to investigate the hypothesis that huntingtin was already involved in development of the nervous system in the last common ancestor of chordates, we isolated and characterised the huntingtin homologue from the amphioxus *Branchiostoma floridae*. In the present paper the amphioxus general term must be referred to *Branchiostoma floridae*.

**Results:**

In this report, we show that the exon-intron organization of the amphioxus huntingtin gene is highly conserved with that of other vertebrates species. The AmphiHtt protein has two glutamine residues in the position of the typical vertebrate polyQ tract. Sequence conservation is greater along the entire length of the protein than in a previously identified *Ciona *huntingtin. The first three N-terminal HEAT repeats are highly conserved in vertebrates and amphioxus, although exon rearrangement has occurred in this region. *AmphiHtt *expression is detectable by in situ hybridization starting from the early neurula stage, where it is found in cells of the neural plate. At later stages, it is retained in the neural compartment but also it appears in limited and well-defined groups of non-neural cells. At subsequent larval stages, *AmphiHtt *expression is detected in the neural tube, with the strongest signal being present in the most anterior part.

**Conclusion:**

The cloning of amphioxus huntingtin allows to infer that the polyQ in huntingtin was already present 540 million years ago and provides a further element for the study of huntingtin function and its evolution along the deuterostome branch.

## Background

Huntingtin is a completely soluble, ubiquitously expressed 350-kDa protein of 3144 aa which, once mutated, causes Huntington's disease (HD), a late-onset neurodegenerative disease characterised by movement disorders, dementia and psychiatric disturbances, and by preferential vulnerability of striatal and cortical neurons [1].

One obviously important portion of the mammalian protein is the polyQ tract, which is present in the normal protein with up to 36 glutamines, but becomes further elongated in the mutant protein as a consequence of the DNA CAG triplet repeat expansion in the gene [1]. The role of the polyQ region in huntingtin's physiological function is currently unknown in mammals. The polyQ tract is found in many transcription factors [2] and, in huntingtin, is followed by a recently discovered polyP tract that may give it structural and biochemical advantages [3]. Recent studies have suggested that the polyP tract helps to maintain protein solubility [4]. It is also possible that, during evolution, an expanded polyQ has conferred important molecular function(s) partially because of its cooperation with the emerging polyP tract. An aberrantly expanded polyQ region in huntingtin is sufficient to cause HD.

Investigating the physiological functions of huntingtin involves a number of difficulties, there is a biological evidence indicating that the protein has individual beneficial activities in the brain (e.g. it is anti-apoptic and neuroprotective) [5]. Its primary amino acid sequence reveals little about its function because it is unlike any other known protein and contains only a few known sequence motifs. However, it does have HEAT repeat consensa (approximately 40-amino-acid-long sequences that occur multiple times) [6,7], whose presence indicates an ability to participate in multiple protein-protein interaction networks, as it has been further documented in subsequent studies [8]. However, the presence of these domains does not allow a complete definition of its biological function(s). Furthermore, the influence of the evolution of the HEAT repeats is far from being established and a more thorough knowledge of their presence in non-vertebrate huntingtin may help us to understand their role in the mammalian protein.

In the absence of information about its three-dimensional structure, comparisons of huntingtin homologues should help to define conserved or newly emergent functional domains in mammalian cells, although only limited information is available about huntingtin in other species. Furthermore, the comparative expression and distribution of huntingtin mRNA in different organisms may be instructive as to its role in mammals. As huntingtin homologues in the vertebrate subphylum are highly conserved, whereas *Drosophila melanogaster *huntingtin diverges substantially (particularly in its N-terminal portion and the absence of the polyQ-rich region) [9], we have recently concentrated our study on the cloning and comparative analysis of invertebrate deuterostome homologues, such as ascidians [10], echinoderms [11; Tartari et al., unpublished] and, as described in this paper, amphioxus. These molecules may share similar (but not identical) functions to those of the human protein and may help in reconstructing the evolution of huntingtin.

Before this study, some of us studied a complete huntingtin gene from the ascidians *C. intestinalis *and *C. savigny *[10] and found that *Ciona *huntingtin contains regions that have specifically evolved in this genus and are concentrated in the central part of the protein, whereas major differences in the N-terminal part indicate the more recent evolution of this group-specific portion of the protein. Furthermore, *C. intestinalis *huntingtin transcript exhibits an alternative splicing in the 3' coding region and in the 3'UTR [10]. One further characteristic of ascidian huntingtin is the complete absence of the polyQ-rich region, whereas polyQ is described for the first time in zebrafish huntingtin that contains a QQQQ tract [12].

A partial huntingtin sequence is available from two sea urchin species [11], which shows that their nervous system organisation is profoundly different from that of chordates [13-15]. *In situ *hybridisation, using a probe from the 3' region of the sea urchin *Heliocidaris erythrogramma *huntingtin homologue, has shown that huntingtin expression is confined to non-neuronal compartments [11]. A similar experiment using the ascidian *Halocynthia roretzi *showed ubiquitous expression of the huntingtin homologue as in vertebrates [11]. On the contrary, vertebrate huntingtin is expressed throughout life and in all tissues, but it is particularly enriched in brain, suggesting that it may play a particular role in this district. Consistently, there is now considerable genetic and biological evidence indicating that huntingtin is important for the formation and maintenance of brain neurons, as it contributes to neuronal survival, neuronal gene expression and BDNF production [16].

Taking advantage of the newly available data from amphioxus *B. floridae *genome sequencing, we here describe the cloning of amphioxus huntingtin (*AmphiHtt*), coming from an invertebrate chordate whose phylogenetic node of divergence is thought to go back 540 million years, while *Ciona *seems to have diverged more recently [17-19]. We also describe for the first time the distribution of huntingtin mRNA in this invertebrate chordate, whose nervous system development is particularly close to that of vertebrates as it includes vertebrate-like anatomical characteristics such as a dorsal nerve cord, a notochord and segmentally arranged muscles.

We show that AmphiHtt protein has two glutamines in the same polyQ tract position of vertebrate homologues, thus suggesting that polyQ was already present 540 million years ago. We also report that the primary sequence around the QQ is highly conserved with respect to vertebrates and that sequence conservation along the entire length of the protein is greater than in *C. intestinalis *huntingtin. The first three N-terminal HEAT repeats are highly conserved in vertebrates and amphioxus, although exon rearrangement has occurred in this region. We also show that amphioxus huntingtin is not exclusively neural, but mainly enriched in the neural compartment; this is a clear indication that huntingtin, in amphioxus, at least within the analyzed developmental stages, could have a specific neuronal function.

## Results

### Cloning and characterisation of the amphioxus huntingtin sequence (*AmphiHtt*)

**Figure 1:**
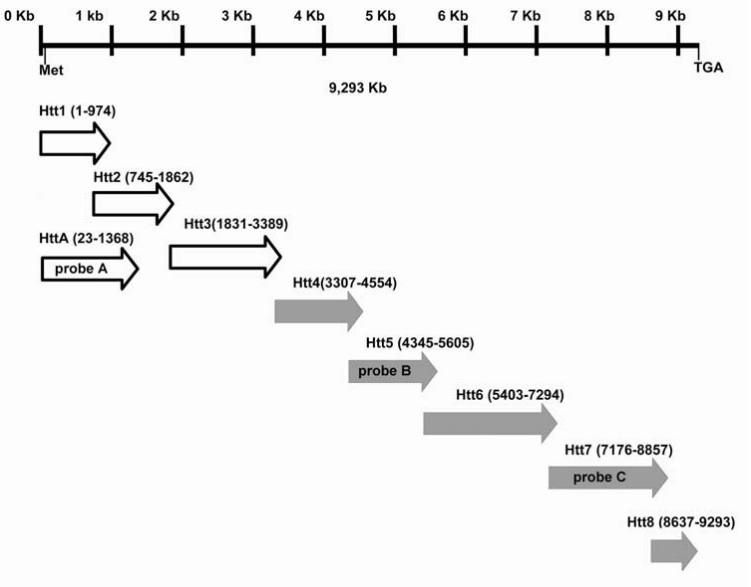
**Schematic representation of the amplification strategy for *AmphiHtt *cDNA isolation**. The coding sequence of *AmphiHtt *is represented by a horizontal line. Four clones were isolated by RT-PCR from an adult amphioxus (empty arrows), and five clones by PCR from an embryonic cDNA library (grey arrows). The numbers in brackets represent the nucleotide position in each fragment. The horizontal lines also show the start of translation (Met) and the stop codon (TGA). The clones used to synthesise the probes are shown as probe A/B/C.

Starting from the recently available *B. floridae *genomic sequencing data, two scaffolds were identified by means of TblastN similarity as possibly containing the huntingtin gene. A first messenger prediction was produced and used to design eight primer pairs (Figure 1 and Additional file 1). PCR assays with first-strand adult cDNA and a 5- to 24-hour *B. floridae *embryos cDNA library as templates, yielded eight overlapping clones that constituted the coding sequence of an amphioxus huntingtin gene, *AmphiHtt*. The *AmphiHtt *cDNA sequence (deposited in GenBank: Accession No. EF210456) is 9293 bp long and contains a putative 2972 bp open reading frame encoding a 3090 amino acid protein; an in-frame stop codon upstream from the putative start codon was found at 9 bp, and a stop codon at 9291 bp.

**Figure 2:**
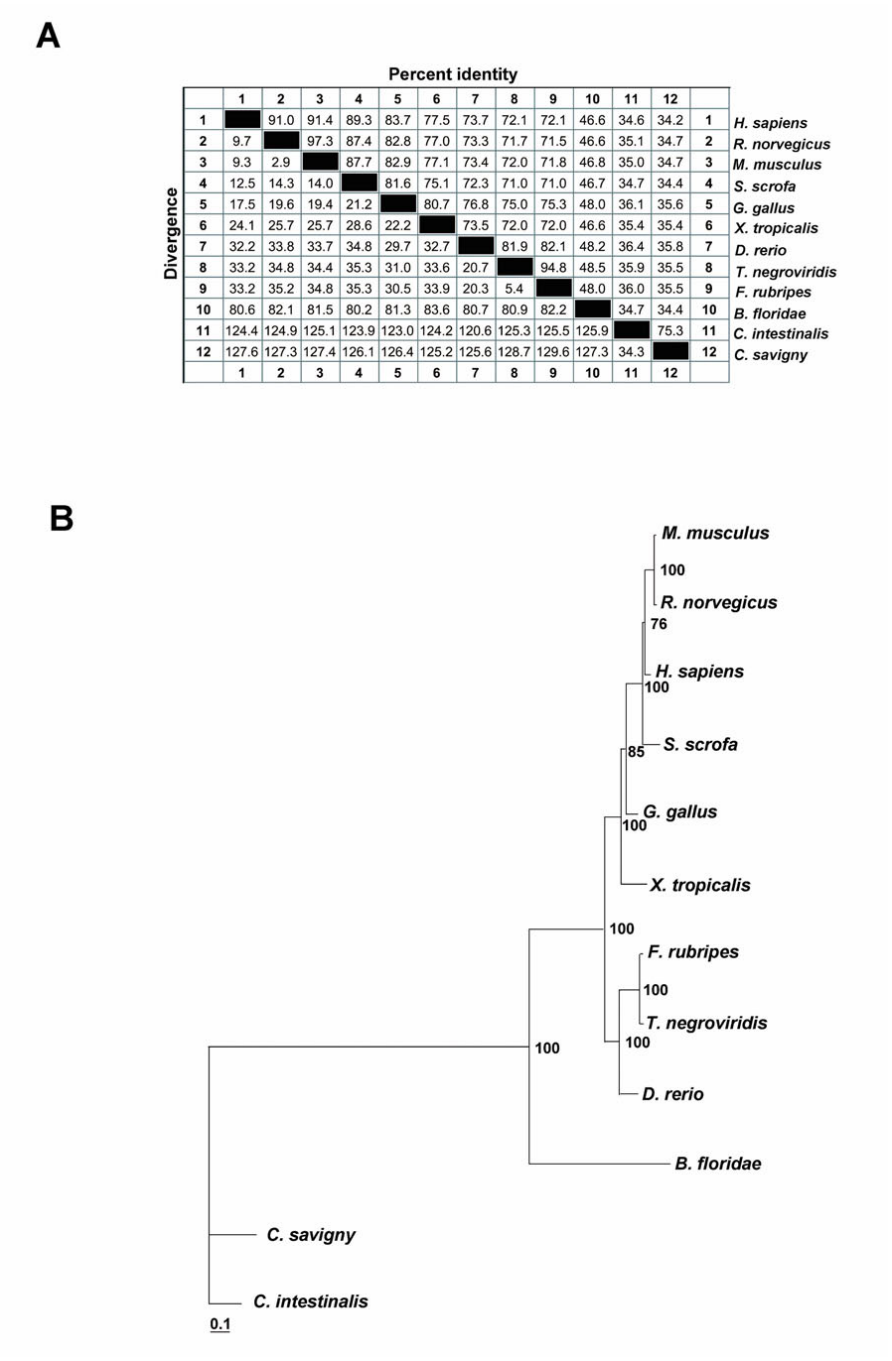
**A. Sequence identity matrix of huntingtin, reconstructed from protein sequences**: percent divergence is calculated by comparing sequence pairs in relation to their relative positions in the alignment; percent identity is estimated by comparing percent sequence identity directly without considering phylogenetic relationships. **B. Unrooted tree of huntingtin proteins. **(The huntingtin proteins used to reconstruct the tree were from the following species: *H. sapiens*,*R. norvegicus*, *M. musculus, S. scrofa*, *G. gallus, X. tropicalis, F. rubripes*, *T. negroviridis*, *D. rerio*, *C. savignyi*, *C. intestinalis, B. floridae*). Phylogenetic analysis reveals that *B. floridae *huntingtin branching just basal to vertebrate huntingtin protein, and that it groups very robustly with vertebrate orthologues. The branch length in the tree is proportional to the number of amino acid substitutions, and the scale bar indicates 0.1 amino acid substitution per position in the sequences. The numbers on each node indicate the percentage confidence values based on 100 replicate bootstrap resamplings of the alignment data.

Sequence similarity analysis of the entire huntingtin sequences of several chordates (Figure 2A) showed that the amphioxus sequence has 46% percent identity with mammals, 46–48% with fish, and 34% with *Ciona*, whereas *Ciona *proteins have only 34–36% identity with vertebrate huntingtin. Furthermore, additional analysis led to the calculation of a 124–127 aa divergence between *Ciona *and vertebrates (Figure 2A), but only an 80–83 aa divergence between amphioxus and vertebrates (Figure 2A). Multiple sequence alignments were generated and huntingtin phylogenetic trees were constructed (Figure 2B, Additional files 2 and 3).

The results obtained using these methodologies indicate that AmphiHtt is more similar to vertebrate huntingtin than to *Ciona *proteins, which apparently conflicts with the current view that tunicates are the sister group of vertebrates [17-19] (probably likely due to the generally high rates of evolution of the tunicate genome [20] with long-branch attraction as a biasing factor in their phylogenetic position).

**Figure 3:**
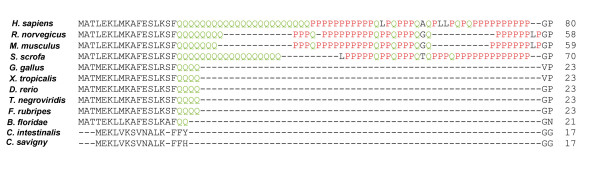
**Sequence comparison of the N-terminus huntingtin domain structure at the level of the glutamine/proline (Q/P)-rich region in different species**. The glutamine residues are outlined in green and the proline residues in red. Detail of polyQ evolution: an increasing trend of inserted glutamine residues from *B. floridae *to *H. sapiens*.

Qualitatively, the AmphiHtt sequence has two glutamines (Q17 and Q18) at the corresponding position to polyQ in vertebrates (Figure 3), whereas *Ciona *huntingtin has an aromatic amino acid group in this position. Amphioxus is therefore the first known non-vertebrate species to contain glutamine residues in huntingtin, thus dating the presence of glutamine in a non-vertebrate contest and indicating that the common ancestor of cephalochordates and vertebrates already possessed this characteristic. The polyP tract is only present in mammalian huntingtin and absent in non-mammalian vertebrates, *Ciona *huntingtin and AmphiHtt. In addition, the first 17 amino acids of AmphiHtt, with its three lysines that have been shown to participate in determining the intracellular distribution of the protein between the cytoplasm and nucleus in vertebrates [21], are also strongly conserved (Figure 3).

Finally, in order to look at a variability in the polyQ tract and at a somatic instability, we carried out a BLAST search of the NCBI amphioxus dbEST and Trace-Archives databases using *AmphiHtt *cDNA sequence as a query. No matching EST sequences were found. Furthermore, all shot gun sequences covering the polyQ tract confirmed the exclusive presence of two glutamines residues and consequently the absence of a somatic instability.

**Figure 4:**
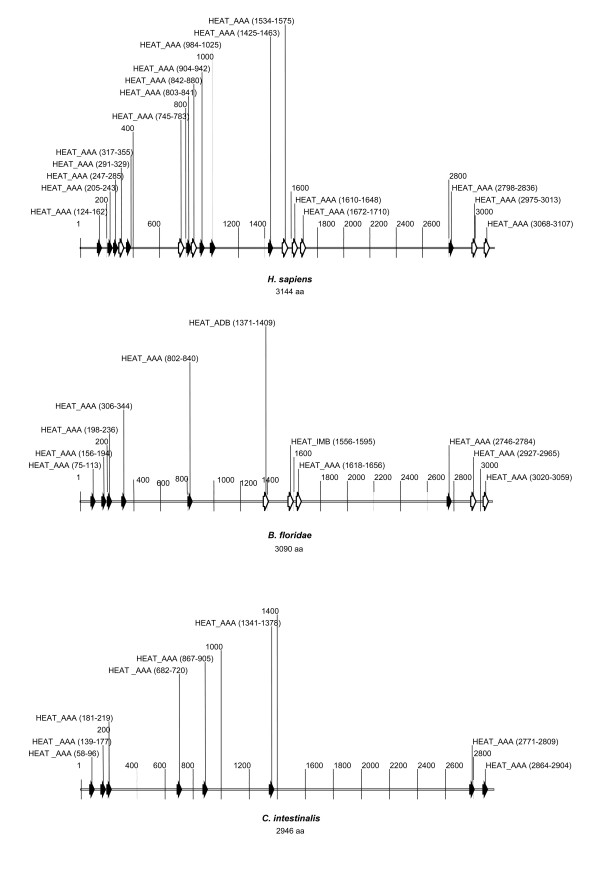
**Comparison of the huntingtin HEAT repeats from *H. sapiens*, *B. floridae *and *C. intestinalis***. Schematic representation of HEAT repeats is drawn to scale. The repeats are indicated as black (high REP scores) and white arrows (low REP scores). The amino acid position of each HEAT repeat is also shown. The sequences relating to the HEAT repeats and their corresponding positions following multiple alignment in the three organisms are shown in Tables 1 and 2.

We next searched for AmphiHtt HEAT repeats by applying the REP program to the AmphiHtt sequence (Figure 4, Tables 1 and 2). HEAT repeats are bioinformatic consensa present in vertebrate huntingtin that may have molecular activity. We therefore applied the score of the consensus, sequence similarity and relative position of the HEATs in a multiple alignment (containing a good number of informative protein sequences) in order to evaluate the most possible functionally active HEATs. Although this evaluation may need to be revised in the future, we found that the program identified six highly scored HEAT-AAA repeats (aa 75–113, aa 156–194, aa 198–236, aa 306–344, aa 802–840, and aa 2476–2784). Comparison of the primary sequences of amphioxus, human and *Ciona *huntingtin revealed five potential additional HEAT repeats (aa 1371–1409, aa 1556–1595, aa 1618–1656, aa 2927–2965, aa 3020–3059) that are consistent with the previously published consensa in human huntingtin [6,7,10], whereas the contrary, none of the consensa specific to the central part of the *Ciona *homologue (aa 867–905 and aa 1341–1378), seems to have a correspondent in amphioxus. Numbering the HEAT consensa of human huntingtin [10] from the N-terminal to the C-terminal end, the sequence and position of the first three amino terminal human HEAT repeats are very well conserved in AmphiHtt (Figure 4, Tables 1 and 2), as are the ninth and eleventh in the central region, and the fifteenth in the C-terminal portion. Amphioxus seems to have one HEAT consensus at aa 306–344 that has no correspondence in human huntingtin, although a similar sequence can be found in *Ciona *huntingtin. An opposite situation can be established for the fourteenth human HEAT, which seems to have a correspondent only in amphioxus and not in *Ciona*, whereas the fifteenth seems to be specifically lost in *Ciona*. Finally, two additional consensa (add1 and add2, see Tables 1 and 2) in the C-terminal portion of *Ciona *huntingtin (aa 2771–2809, aa 2864–2904) met a possible correspondence in both amphioxus (aa 2927–2965, aa 3020–3059) and human huntingtin.

**Table 1:** HEAT repeat sequence list

	***H. sapiens *consensus**	**aa position**		***B. floridae *consensus**	**aa position**		***C. intestinalis *consensus**	**aa position**
**1**	HEAT_AAA	124–162	**1**	HEAT_AAA	75–113	**1**	HEAT_AAA	58–96
**2**	HEAT_AAA	205–243	**2**	HEAT_AAA	156–194	**2**	HEAT_AAA	139–177
**3**	HEAT_AAA	247–285	**3**	HEAT_AAA	198–236	**3**	HEAT_AAA	181–219
**4**	HEAT_AAA	291–329						
**5**	HEAT_AAA	317–355						
			**4**	HEAT_AAA	306–344			
**6**	HEAT_AAA	745–783						
**7**	HEAT_AAA	803–841						
**8**	HEAT_AAA	842–880						
**9**	HEAT_AAA	904–942	**5**	HEAT_AAA	802–840	**4**	HEAT_AAA	682–720
**10**	HEAT_AAA	984–1025						
						**5**	HEAT_AAA	867–905
**11**	HEAT_AAA	1425–1463	**6**	HEAT_ADB	1371–1409			
**12**	HEAT_AAA	1534–1575				**6**	HEAT_AAA	1341–1378
**13**	HEAT_AAA	1610–1648	**7**	HEAT_IMB	1556–1595			
**14**	HEAT_AAA	1672–1710	**8**	HEAT_AAA	1618–1656			
**15**	HEAT_AAA	2798–2836	**9**	HEAT_AAA	2746–2784			
**add1**	HEAT_AAA	2975–3013	**add1**	HEAT_AAA	2927–2965	**7**	HEAT_AAA	2771–2809
**add2**	HEAT_AAA	3068–3107	**add2**	HEAT_AAA	3020–3059	**8**	HEAT_AAA	2864–2904

From top to bottom: list of HEAT repeats in *H. sapiens*, *B. floridae *and *C. intestinalis *huntingtin from N-terminus to C-terminus, with the corresponding amino acid position. Reading the panel from left to right, the corresponding HEAT repeats in the three organisms are on the same line.

**Table 2:** HEAT repeat consensus sequence

	**consensus**		**sequence**
**1**	***H. sapiens***	HEAT_AAA	QKLLGIAMELFLLCSDDAESDVRMVADECLNKVIKALMD
	***B. floridae***	HEAT_AAA	PKFLGISMEMFLASCDDKESDVRMVADECLNRTVKMLLE
	***C. intestinalis***	HEAT_AAA	PGLLAVSVETLLQSCADDNADVRLNANECLNRLIKGLYE
**2**	***H. sapiens***	HEAT_AAA	RPYLVNLLPCLTRTSKRPEESVQETLAAAVPKIMASFGN
	***B. floridae***	HEAT_AAA	RPYVVNLLPCFNRICRRQEDAVQETLANSLKKTFPVLGS
	***C. intestinalis***	HEAT_AAA	RPYILNLLPCLCRISQREEDGVQETLGLSLVKIFKILGP
**3**	***H. sapiens***	HEAT_AAA	DNEIKVLLKAFIANLKSSSPTIRRTAAGSAVSICQHSRR
	***B. floridae***	HEAT_AAA	DAEIKVLLKTFLPNLRSASAVTRRTAASSLVTFCQHSRK
	***C. intestinalis***	HEAT_AAA	ESEIQGLLASFLKNLSHKSATMRRTACVCLHSVILNCRK
**9**	***H. sapiens***	HEAT_AAA	KLQERVLNNVVIHLLGDEDPRVRHVAAASLIRLVPKLFY
	***B. floridae***	HEAT_AAA	NCQQRLLEDIVLHLMGDDDYRVRHAATAALVRLVPRLFY
**11**	***H. sapiens***	HEAT_AAA	RLFEPLVIKALKQYTTTTCVQLQKQVLDLLAQLVQLRVN
	***B. floridae***	HEAT_AAA	RLFEPLVIKSLKLYTVTSSVTLQRQVLHLLAQLVQLRVN
**15**	***H. sapiens***	HEAT_AAA	DDTAKQLIPVISDYLLSNLKGIAHCVNIHSQQHVLVMCA
	***B. floridae***	HEAT_AAA	SEATKLLVPVLQDYLSKNIPPTAQCCIVHVEPHVLAMWA
**add1**	***H. sapiens***	HEAT_AAA	ARVVARILPQFLDDFFPPQDIMNKVIGEFLSNQQPYPQF
	***B. floridae***	HEAT_AAA	ARVVARILPTFLDDFFPAQDIMNKVIGEFLSSQQPHPQL
	***C. intestinalis***	HEAT_AAA	ARVMSKVLPSMLDDFFPAQDIMNKIIAEFISTLQPFPAS
**add2**	***H. sapiens***	HEAT_AAA	SPWVAAILPHVISRMGKLEQVDVNLFCLVATDFYRHQIEE
	***B. floridae***	HEAT_AAA	NPWVCALLPHVIGRMGLMETVDRKLFCITALDFYKNQITE
	***C. intestinalis***	HEAT_AAA	NRWISSMVPLIISRVHDPTLDVDWTCFCKAAVDFYTCQLSE

The consensus sequence of the most conserved HEAT repeats in *H. sapiens*, *B. floridae *and *C. intestinalis *huntingtin are shown.

### Genomic organisation of the huntingtin gene: a comparative overview

**Figure 5:**
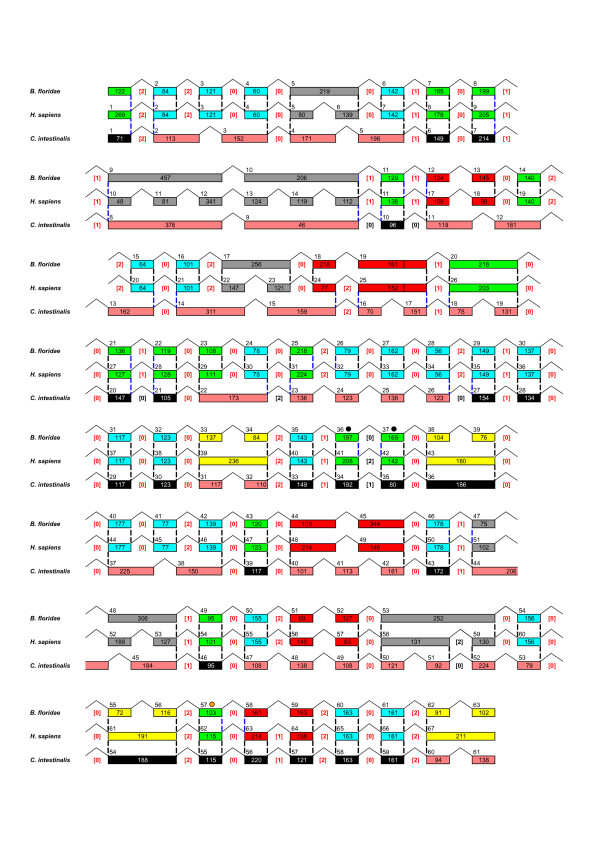
**Genomic organisation of the huntingtin gene from *B. floridae*, *H. sapiens *and *C. intestinalis***. Introns are represented by broken lines and exons by coloured boxes. All of the corresponding introns are indicated as black/blue dashed lines. The base-pair length of each exon is indicated inside the boxes. The numbers above the coloured boxes represent the exon numbering. Bracketed red numbers correspond to the common intron phase; the different intron phase is indicated by bracketed black numbers. Black dots label the exons coming from scaffold_613 and an orange dot shows the exon deduced from scaffold_378. Only the protein coding sequence was considered for the reconstruction of the genomic organisation. The diagram is not drawn to scale. See text for further details.

The *AmphiHtt *cDNA sequence was superimposed on the genomic sequence available at JGI, and it was found that the genomic coordinates for the *AmphiHtt *cDNAs were from 29810 nt to 81840 nt in scaffold_613 (minus strand), and from 685048 nt to 743806 nt in scaffold_378 (plus strand). Taking advantage of the newly cloned cDNA sequence, we reconstructed the genomic organisation of the huntingtin gene in both scaffolds using two genomic mapping software programs: GMAP and Wise2. The amphioxus huntingtin gene contains 63 exons and spans a genomic region of over 50 kb, whereas vertebrate sequences have 67 exons and corresponding gene lengths ranging from 80 Kb (fishes) to 180 Kb (humans), and *Ciona *has 61 coding exons covering a genomic region of 33 kb [10]. The predicted sequence of our *AmphiHtt *corresponded with minor polymorphisms to the coding sequences predicted from the two genomic scaffolds. Nevertheless, as the two scaffolds have some assembly errors and several tandem repeat elements (deduced using the Tandem repeats Finder program, [22]), the information on exons 36 and 37 comes from the scaffold_613, and that on exon 57 from the scaffold_378 (Figure 5 and Additional file 4). Furthermore, in this preliminary genomic assembly, the information in some intron sequences is not conclusive but seems to match our exon mRNA data perfectly: 60 exons are correctly recognised in both scaffolds. We therefore suggest that there is only a single copy gene of huntingtin in the amphioxus genome, and that the two scaffolds represent the two alleles of the same gene.

Comparison of the genomic and cDNA sequences of *AmphiHtt *allowed us to determine its exon/intron structure, and to compare it to what is known for members of the same family in other chordates. Furthermore, analysis of the pattern of exon-intron junctions can provide important insights into the evolution of huntingtin genes. In particular, as shown in Figure 5, we compared the genomic organisation of the *H. sapiens *(Chr4:3103557–3288752; assembly version v35), *B. floridae *and *C. intestinalis *(scaffold_31: 333864–386142; assembly version v 1.95) huntingtin genes. Comparison of the conservation of the exon/intron boundaries revealed the presence in amphioxus of 51 introns in conserved positions, including 43 completely conserved introns (dashed lines outlined in black in Figure 5) and eight that are not in exactly the same position but have slipped of 4–18 bp (dashed lines outlined in blue in Figure 5). In addition, there are 38 orthologous exons in which the predicted amino acid sequences from amphioxus and *H. sapiens *can be aligned over the entire length: 14 of different lengths and 24 of identical length (respectively shown as green and blue boxes in Figure 5). The other exons are grouped in 14 exon-clusters defined as groups of exons delimited by introns that are positionally conserved or which have slipped by 4–18 bp (dashed lines outlined in blue in Figure 5), five of which are indicated as red box when the number of exons is the same in both species; whereas the other five exon-clusters have more exons in *Homo sapiens *than in amphioxus (grey boxes), and the remaining four show the opposite situation (yellow boxes) (Figure 5). Otherwise, *Ciona *has 23 orthologous exons (black boxes) and 17 exon-clusters (pink boxes) (Figure 5).

The *AmphiHtt *gene has a highly conserved distribution of exons and introns with respect to the human sequence (Figure 5), and a length range of 60–457 bp that does not substantially differ from that of the human gene (48–341 bp). The exon/intron splice sites in *AmphiHtt *correspond to the expected GT-AG intron consensus splicing sequences; the intron phases in *AmphiHtt *are identical to those in the human gene with the exception of intron 36 (Figure 5); and the majority of introns are in phase 0. In particular, the *AmphiHtt *gene shares with the human huntingtin gene 28 phase 0, 10 phase 1 and 12 phase 2 introns, thus indicating a more conserved gene structure than the *Ciona *gene homologue, that has 18 phase 0, 8 phase 1 and 6 phase 2 introns (Figure 5).

In conclusion, on the basis of such analysis, we found that: i) the exon-intron organisation of the huntingtin gene is remarkably conserved in the phylum Chordata, as both amphioxus and *Ciona *huntingtin genes have a very similar genomic organisation to that of other vertebrate species; ii) at least four reduction events in exon numbers (yellow exon-clusters) occurred between the amphioxus and human genes, which are preferentially located at the 3' end and in the central region of the gene, whereas greater exon acquisition has mainly occurred in the 5' end. This confirms previous observations that the greater exon acquisition corresponds to a larger difference in the N-terminal part of the protein between human and *Ciona *huntingtin [9]. However, the genomic organisation of the amphioxus huntingtin gene is more similar to that of vertebrates than to *Ciona*, including the larger number of positionally conserved introns (51 in amphioxus against 39 in *Ciona*), the smaller number of exon-clusters (14 in amphioxus against 17 in *Ciona*), and the conservation of intron phases. These differences could be also explained by a high evolutionary rate such as that observed in tunicate species.

### *AmphiHtt *expression in amphioxus

**Figure 6:**
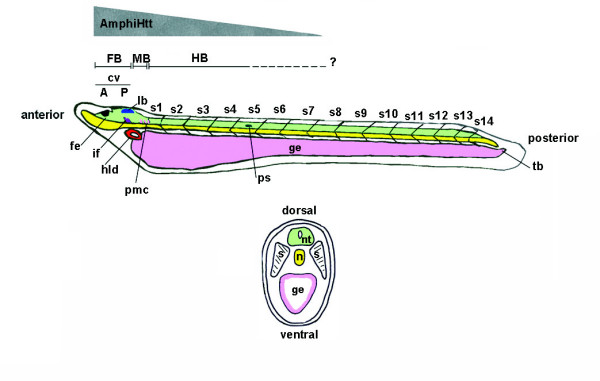
**Schematic diagram of a *B. floridae *larva showing some of the details useful for following *AmphiHtt *expression. Upper panel**: Longitudinal view of an amphioxus larva (green: nervous system; yellow: notochord; pink: gut endoderm). The central nervous system has homologues of at least three major vertebrate brain subdivisions: the fore-, mid-, and hindbrain. The cerebral vesicle (cv) can be subdivided into rostral (A, anterior) and caudal (P, posterior) parts, and only corresponds to the diencephalic region of the vertebrate forebrain (FB) because it lacks of a telencephalic region [43]. The anterior part of the cerebral vesicle consists of the frontal eye complex (fe), characterised by a cluster of pigmented epithelial cells (pigment cup) and four rows of neurons [44]. The posterior part begins with the infundibular cells (if) that are responsible for producing Reissner's fibres in amphioxus and it is homologous to a vertebrate diencephalic region corresponding to the subcommisural organ [45]. Beginning near the front of the posterior cerebral vesicle, the lamellar body (lb), a putative homologue of the vertebrate pineal eye (epiphysis) [43], is located dorsally. This is followed by a midbrain-like region (MB), including the tectal zone extending from the posterior part of the lamellar body to the anterior part of the primary motor centre (pmc), and then a hindbrain-like region (HB), that starts from the posterior part of the pmc and extends caudally over the first pigment spot (ps), has an uncertain posterior limit (?) [46,47]. Some non neuronal structures are indicated: hld, Hatchek's left diverticulum; s, somites; tb, tail-bud. A grey triangle indicates the expression gradient of *AmphiHtt *from the most anterior part to approximately two-thirds of the neural tube. **Lower panel**: Median cross section of an amphioxus larval body showing the dorso-ventral organisation of the major structures: nt, neural tube; n, notochord; e, endoderm; s, somites. The neural tube is positioned dorsally to the notochord (n) and somites (s) are positioned laterally to the neural tube (nt).

Analyses of huntingtin expression in vertebrates have provided limited information concerning its potential physiological function as the protein is expressed ubiquitously and throughout the entire life of a vertebrate. A first attempt to evaluate huntingtin distribution in an invertebrate organism was made by Kauffman *et al*. [11], and we tested the expression of *AmphiHtt *mRNA during amphioxus development. Amphioxus and vertebrates share anatomical features such as a dorsal nerve cord, a notochord, segmentally arranged muscles (myomeres), pharyngeal gill slits and a post-anal tail (see Figure 6).

We performed whole mount *in situ *hybridisation on *B. floridae *developmental stages of 0–10 hours, 11-hour early neurula, 15-hour late neurula, 18-hour late neurula, 24-hour early larva, and 48-hour larva. In order to increase our confidence in the results (the presence of possible alternative transcripts that may be differentially expressed and that are not identified in the present work) we used three different >1000 bp probes mapping to the 5', central and 3' portions of the messengers. We obtained the same results using both mixed probes and one probe at a time in separate experiments.

**Figure 7:**
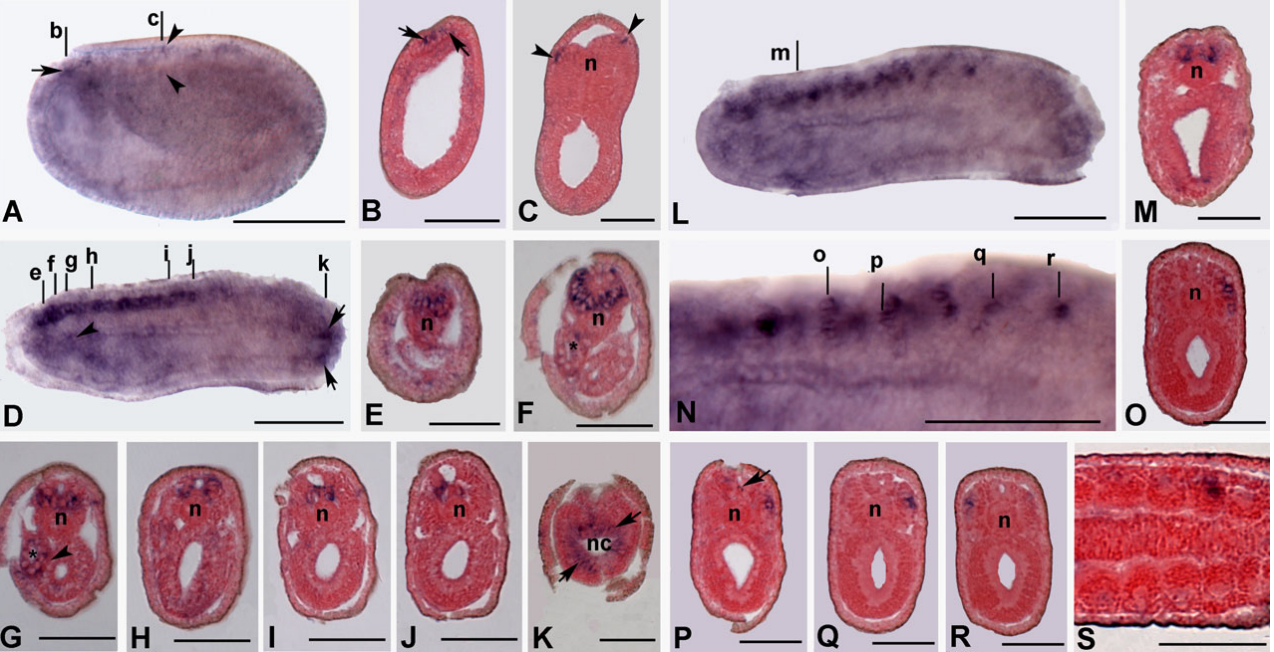
**Developmental expression of *AmphiHtt *in *B. floridae *neurulae**. In the whole mounts, anterior is on the left; the cross sections are counterstained in pink and viewed from the posterior end of the animal. Scale lines for whole mounts = 50 μm and for sections = 25 μm. **A**. Side view of 11-hour neurula with *AmphiHtt *expression in the most anterior neural plate (arrow), and in some cells of the neural folds (arrowheads). **B, C. **Cross sections through levels b and c in A. Transcripts are visible in the most anterior tip (arrows) and at the edges (arrowheads) of the neural plate. **D. **Side view of 15-hour neurula with *AmphiHtt *expression in the cerebral vesicle and the most anterior two-thirds of the neural tube. Some labelled cells are also visible in Hatschek's left diverticulum (arrowhead) and in cells of the neuroenteric canal (arrows). **E-J**. Cross sections through levels e-j in D. Strong neural expression was found in the cerebral vesicle (E-G) and ventrolateral neurons of the neural tube (H-J). *AmphiHtt *is also expressed in some cells of Hatschek's left diverticulum (arrowhead) (G). **K. **Cross sections through level k in D showing expression in the endoderm of the neuroenteric canal (arrows). **L. **Side view of 18-hour neurula: *AmphiHtt *expression is mainly localised at somite level and in the neural tube. **M. **Cross-sections through level m in L show details of the transcript localisation in a pair of ventrolateral neurons in the neural tube. **N. **High-resolution images of the preceding specimens throughout somites 3–10. **O-R **Cross-sections through levels o-r in N showing *AmphiHtt *expression in groups of cells located dorsolaterally in the somites. The arrow in P indicates the primary pigment spot in the nerve cord. **S**. Frontal section of 18-hour neurula showing two labelled cell bodies located near the somite boundaries. Abbreviations: n, notochord; asterisks, Haschek's left diverticulum; nc, neuroenteric canal.

No detectable transcripts of *AmphiHtt *were found between fertilisation and gastrula stage (0–10 hrs) (data not shown). The first visible expression was found at the most anterior neural plate of 11-hour early neurula (Figure 7A). At this stage, the *AmphiHtt *transcripts are mainly located at the anterior tip of the neural plate (Figures 7A and 7B), and in some more posterior cells at the neural plate borders (Figures 7A and 7C). As neurulation proceeds and the neural tube forms by the dorsal folding of the lateral edges of the neural plate, *AmphiHtt *expression extends along the antero-posterior axis of 15-hour neurula. At this stage, neural expression is found in the entire cerebral vesicle and in the most anterior two-thirds of the neural tube (Figure 7D).

**Figure 8:**
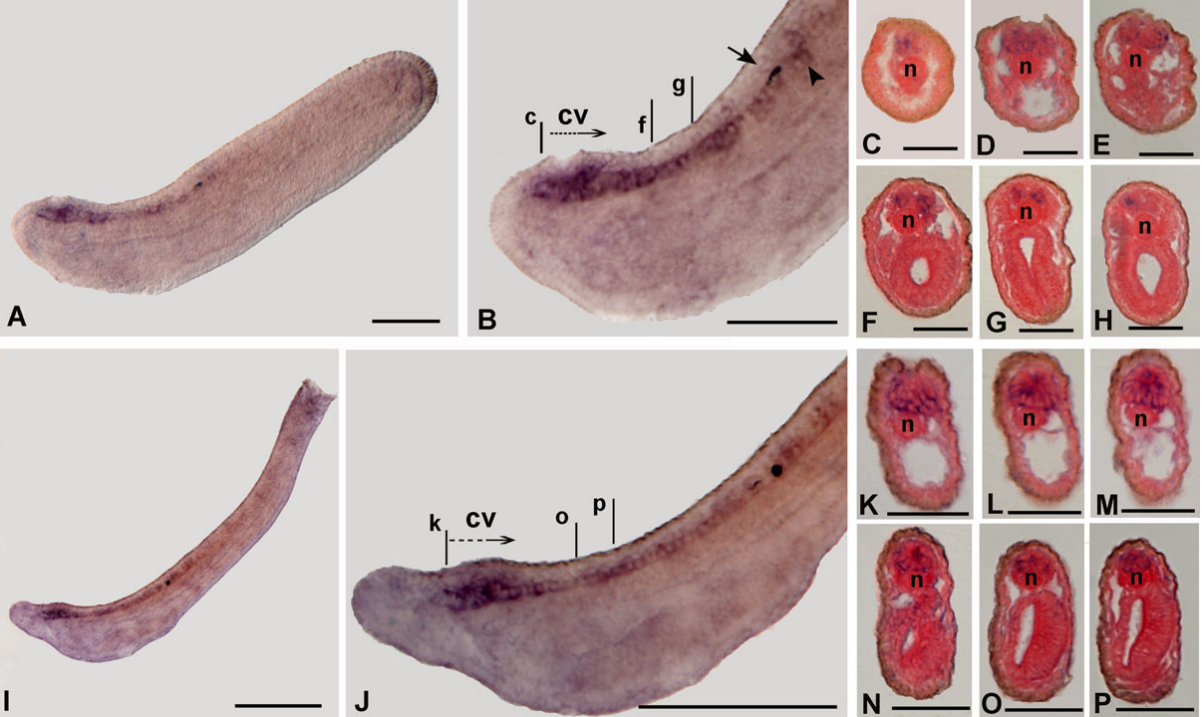
**Developmental expression of *AmphiHtt *in *B. floridae *larvae**. In the whole mounts, anterior is on the left; cross sections are viewed from the posterior end of the animal. Scale lines for whole mounts = 50 μm and for cross-sections = 25 μm. **A. **Side view of 24-hour larva with *AmphiHtt *expression in the neural tube. **B. **Enlargement of the most anterior part of the preceding specimens showing conspicuous expression in the cerebral vesicle (cv) and anterior neural tube until the first pigment spot (arrow). Some positive neural cells (arrowhead) were also found just behind the first pigment spot. **C-E. **Consecutive cross-sections starting from c and proceeding along the dotted arrow shown in B. A positive signal is present in neurons of the cerebral vesicle. **F,G. **Cross-sections through levels f and g in B, showing a pair of specifically labelled ventrolateral neurons. **H. **Cross-section through the level shown by the arrowhead in B. *AmphiHtt *is expressed in a single ventrolateral neuron. **I. **Side view of 48-hour larva. *AmphiHtt *expression is only visible at the level of neural tube. **J. **High-resolution image of two-thirds of the preceding specimen. The *AmphiHtt *transcript is conspicuously present in the cerebral vesicle and neural tube. **K-N. **Cross-sections starting from k and proceeding along the dotted arrow shown in J. Labelled neurons were found in the developing frontal eye complex (K-M). Transcripts were also visible at the level of ventral infundibular cells (N). **O, P. **Cross-sections through the levels o and p shown in J. *AmphiHtt *is expressed in some ventrolateral neurons of the nerve cord. Abbreviations: n, notochord; cv, cerebral vesicle.

In order to reveal differences in dorso-ventral distribution, we used cross-sections of the same embryo (15-hour neurula) and found transcripts in some ventrolateral (Figures 7E–G) and dorsolateral cells of the cerebral vesicle (Figure 7F), at the level of the precursor of the frontal eye complex and the infundibular organ. More posteriorly, we found labelled ventrolateral nerve cells of the hindbrain (Figures 7H–J), most of which consisted of paired neural cells located ventrolaterally in the neural tube and may correspond to differentiating DC motoneurons that innervate the dorsal compartment of the myomeres [23,24]. Furthermore, at the 15-hour stage (early neurula), non-neural expression appears in some endodermal cells of the tail bud around the neuroenteric canal (Figures 7D and 7K), and in some cells of Hatschek's left diverticulum (Figures 7D and 7G). Neural tube expression is strongly maintained in late neurulae (18 hours), and new labelling appears in individual somite cells, which were only detected at the 18-hour late neurula stage, mainly confined between somite 3 and somite 10, and sometimes arranged as a row of cells at the most lateral margins of the somites, just near the epidermic layer (Figures 7N–S). At the 24-hour early larval stage, the expression was found in the neural tube (Figures 8A–H), being localised to some cells of the cerebral vesicle (Figures 8A–E) and cells of the most anterior two-thirds of the neural tube (Figures 8A,B,F,G). *AmphiHtt*-expressing cells can also be seen in the ventro-lateral position of the neural tube, just behind the first pigment spot (Figure 8H). This pattern of expression is essentially maintained in the later stages of development (48-hour larva) (Figures 8I–P), but the highest expression of *AmphiHtt *mRNA is found at the level of the cerebral vesicle (Figures 8J–M).

In conclusion, our findings demonstrate that during amphioxus development huntingtin transcripts are detected into the neuronal compartment starting from early neurula stage. Except for endodermal (tail bud) and mesodermal structures (Hatschek's left diverticulum and somites) (i.e. non neuronal cells in the 15- to 18-hour stages), the expression of *AmphiHtt *mRNA follows an antero-posterior gradient, and is enriched in the anterior neural tube. This result could reflect a specific neuronal function of huntingtin in the middle and later developmental stages of this non-vertebrate organism. Nevertheless, we cannot exclude that in situ hybridization, being a relatively insensitive techniques, do not allow us to detect low levels of messengers in the early developmental stages and in non nervous structures of amphioxus larvae.

## Discussion

In order to increase our understanding of normal huntingtin function(s) and reconstruct polyQ evolution along the deuterostome branch as an indication of possible protein activity, we have recently concentrated our study on the cloning and comparative analysis of non-vertebrate deuterostome homologues, such as ascidians [10], echinoderms (Tartari et al., unpublished) and amphioxus. Amphioxus shares the full suite of chordate characteristics with vertebrates and the nerve cord has dorso-ventral specialisation, but they lack the vertebrate typically extensive subcellular and tissue specialisation of the nervous system. At genetic level, amphioxus did not undergo the extensive gene duplication events that characterise vertebrate genomes [25,26], possibly lacking the newly-acquired gene innovation of vertebrates. All of these characteristics make this organism particularly useful to infer features that were already present in the last common ancestor of chordates.

Along the deuterostome branch, the recently cloned ascidian huntingtin homologue [10], whose sequence conservation is greater than that of *Drosophila*, suggests a more recent evolution of the 5' end of the gene, which is also characterised by the lack of a polyQ tract. Only partial sequences are available from other invertebrates in the deuterostome branch, such the tunicate *Halocynthia roretzi*, and from two echinoderms, *Strongylocentrotus purpuratus *and *Heliocidaris erythrogramma*, for which no extensive details of sequence conservation are known [11].

Huntingtin cloning from amphioxus allowed us to discover an evolutionarily ancient point of emergence of the polyQ tract similar to that characterising huntingtin in vertebrates. AmphiHtt protein has two glutamines in the same position of the polyQ tract as that characterising the entire vertebrate subphylum. This indicates that a double Q was present in last common ancestor between cephalochordates and vertebrates, and that C*iona *has differently and subsequently lost this characteristic.

A further biochemical indication of the possible molecular activity of the protein is the presence of HEAT repeat consensa. The AmphiHtt protein has 11 HEAT repeats, thus falling between the 8 of *C. intestinalis *and the 17 of human huntingtin. Our analyses identified the most conserved HEATs (the first three in the N-terminus, the ninth and eleventh in the central region, and the fifteenth in the C-terminus) in a homologue that precedes vertebrate genome duplication. Although this does not yet allow us to confirm a HEAT repeat-dependent evolutionary trend in huntingtin, or the impact of these sequences on protein function, we can report the strong maintenance of HEATs at the extreme N-terminus.

With respect to human huntingtin, conservation of the primary amino acid sequence in the remaining amphioxus protein is greater than that in *C. intestinalis*, and comparison of the gene structure of *AmphiHtt *and the human and ascidian homologues shows that the gene and exon boundaries are more conserved with respect to vertebrates than the ascidian huntingtin gene. This suggests that amphioxus huntingtin is closer to, and less divergent from vertebrate huntingtin than ascidian huntingtin, and leads us to hypothesise that its function is possibly also closer to that of vertebrate huntingtin.

In particular, as exon acquisition events are mainly located in the 5' portion of the gene, whereas the extreme N-terminal portion of AmphiHtt is highly conserved at protein level, we suggest that huntingtin refined its possible N-terminal corresponding function in the evolutionary transition between cephalochordates and vertebrates, and we postulate that this function can be linked to the emergence of a role of huntingtin in the nervous system (at least during development) as amphioxus huntingtin messenger RNA is enriched in neuronal tissues.

First expression analysis on deuterostome invertebrates the echinoderm *H. erythrogramma *and the ascidian *H. roretzi *[11], suggests an ubiquitous expression of huntingtin mRNA at all developmental stages of ascidian (as in vertebrates), and a non-neuronal signal in echinoderms. Moreover, by RT-PCR *Drosophila *huntingtin transcripts were found in all developmental stages [9].

The complete cloning of *AmphiHtt *also allowed us to analyse its expression in the embryonic and larval stages of amphioxus, and may help in inferring hypothesis on possible huntingtin function.

The pattern of huntingtin expression in amphioxus substantially differs from that at the corresponding stages of vertebrate development. It is mainly limited to the neuronal compartments from 11-hour neurula to 48-hour larva, and is not detectable at the early developmental stages until the gastrula stage, whereas it seems to be widely expressed in vertebrates. In humans, rodents and pigs, huntingtin is ubiquitously expressed but has the highest levels in brain and testis, followed by lung, heart, kidney and liver. Even lower vertebrates (fish) seem to express huntingtin at all developmental stages and in all tissues, particularly in the head of adults [12].

Huntingtin does not seem to be expressed until the end of gastrulation in amphioxus. Although this finding cannot exclude the possibility of simply undetectable low messenger levels, it is possible that, unlike in vertebrates, huntingtin may not be required for gastrulation in this organism. Mammalian data indicate that huntingtin is required at different stages of development, and that its total absence causes embryo lethality at the gastrulation stage [27-29]. However, amphioxus embryos differ from mammalian embryos in early gastrulation insofar as they have a double-layered gastrula (ectoderm and meso-endoderm) instead of the three-layered vertebrate gastrula (ectoderm, mesoderm and endoderm) [30].

As mammalian embryogenesis proceeds, huntingtin is required for epiblast formation and neurogenesis [31]. Finally, the removal of huntingtin from post-natal neurons causes cell death, which indicates that mammalian huntingtin plays an important role in nervous system formation and neuronal survival in adulthood. In any case, the expression of mammalian huntingtin is always ubiquitous at all these stages of development.

Closer analysis of amphioxus huntingtin expression in the nervous system shows an initial homogeneous localisation in the most anterior two-thirds of the neural tube, where the signal seems to become more intense at later stages of development [48 hours), thus indicating a possible antero-posterior gradient. This suggests that amphioxus huntingtin may play a role in events occurring at the time of neurogenesis.

In addition, serial cross-sections of whole-mounted labelled amphioxus embryos, showing dorso-ventral views of specific neural tube regions, revealed the presence of huntingtin throughout the most anterior cerebral vesicle, whereas it was restricted ventrally to the posterior cerebral vesicle. Moving caudally, huntingtin specifically marks some paired ventro-lateral cells in the hindbrain that can be assumed to be dorsal compartment (DC) motor neurons and, even more caudally (after the first pigment spot), huntingtin transcripts preferentially localise dorso-laterally in the neural tube. This is the first evidence of the preferential sub-regionalisation of huntingtin expression in the nervous system.

## Conclusion

We have recently hypothesised that the different functions of huntingtin during mammalian development may possibly reflect evolutionary steps in the protein and that its early non-neuronal activity in mammals can be likened to its ancestral function in species with a poorly organised or no nervous system [5]. In this study, we found that the sequence of amphioxus huntingtin is not critically different from that of vertebrates, and that its expression is particularly enriched in the nervous system. In this view, it can be inferred that an ancestral neuronal function of huntingtin was present 540 millions years ago. The differences in the length of the polyQ tract between amphioxus and vertebrates suggest that the function of huntingtin may have evolved different biochemical properties in both lineages. In particular, we argue that the domain(s) involved in these ancestral function(s) are positioned in the extreme N-terminal portion as the protein's primary sequence and the consensa of secondary structures (HEAT repeats) are highly conserved with respect to vertebrate huntingtin, and because the corresponding 5' portion of the gene seems to be due to more recent evolution.

## Methods

### Animal collection and RNA preparation

Ripe specimens of the Florida amphioxus (*Branchiostoma floridae*) were collected in Old Tampa Bay, FL. Animals were induced to spawn by electric stimulation. Eggs obtained from electrically stimulated females were fertilized, and the developmental stages were raised in laboratory culture. Adult specimens were harvested and immediately submerged in RNA later (Ambion Europe Ltd., UK). Total RNA from a single adult was extracted using the TRIzol LS reagent (Invitrogen, San Diego, CA). Following extraction, RNA was treated with RNAse-free DNAse I (Ambion Europe Ltd., UK) according to the manufacturer's recommendations in order to digest contaminating genomic DNA. First-strand cDNA was synthesised with 5 μg RNA using the SuperScript first-strand synthesis system (Invitrogen, San Diego, CA) and oligo(dT) primers.

### Retrieving sequence from the *Branchiostoma floridae *genome

The *B. floridae *genome assembly (v1.0) was searched at JGI [32] using the TblastN algorithm and several vertebrate huntingtin protein sequences as queries. The identified sequences were analysed by means of two gene prediction programs (GenomeScan [33], GENSCAN [34]) in order to correct the preliminary annotation reported at JGI (Protein ID: 101261, 101262 and 252341). Then, a predicted coding sequence for the amphioxus huntingtin gene was used to define the PCR amplification strategy (Figure 1). Finally, we reconstructed the genomic organisation of the amphioxus huntingtin gene using two genomic mapping software programs: GMAP [35] and Wise2 [36], which respectively re-align messengers and protein to genomic sequences.

### Cloning of *AmphiHtt *mRNA

The resulting first-strand cDNA of adult amphioxus *B. floridae *and a 5- to 24-hour *B. floridae *embryo cDNA library (kindly provided by Jim Langeland) were used in PCR assays with specific primers designed on the basis of the predicted coding sequence. PCRs were carried out in a 50 μl reaction mixture using the Hot Master mix in accordance with the manufacturer's instructions (Eppendorf Srl, Italy) and the primers specified in Additional file 1. The PCR products (Figure 1) were directly cloned using a TOPO TA cloning kit (Invitrogen, San Diego, CA). Ten clones for each amplified fragment were randomly chosen for automated sequencing using a 377 PerkinElmer sequencer and the universal or internal sequence-specific primers.

### Sequence and phylogenetic analysis

The Vector NTI Suite (version 9.0, Informax, North Bethesda, MD) software package was used for sequence analysis. Multiple sequence alignments were carried out using the huntingtin sequences from *Homo sapiens *(P42858), *Mus musculus *(P42859), *Rattus norvegicus *(P51111), *Sus scrofa *(BAA36752), *Danio rerio *(AAC63983), *Fugu rubripes *(P51112), *Tetraodon negroviridis *(CAG03293), and *Ciona intestinalis *(AM162277). We also used the sequences of *Xenopus tropicalis*, *Gallus gallus *and *Ciona savigny *predicted from genomic sequences by Gissi *et al*. [10]. The amino acid sequences from amphioxus and eleven other species were aligned using the CLUSTAL W program [37] and manually corrected. Amino acid sites with gaps in any sequence were excluded, and so a total of 2491 characters were considered for the analysis. The best-fitting model of evolution (JTT, with an estimated alpha parameter to 0.73 and a gamma distribution of rates between sites of 4.0) was inferred by means of the ProtTest [38]. Phylogenetic analysis was performed using a fast and accurate maximum likelihood heuristic method (PHYML v2.4.4) [39] starting from the BIONJ tree, under the parameters estimated by ProtTest. Tree stability was assessed by means of a bootstrap analysis with 100 cycles. Phylogenetic analysis was also performed by CLUSTAL W program and MEGA version 3.1 [40] (Additional file 3). The tree was produced using the neighbor-joining method with Poisson correction and complete deletion of gaps and bootstrapped 1000 times. Such tree was rooted using the huntingtin from *D. melanogaster *(AF146362) as the outgroup. The phylogenetic trees were visualised using TREEVIEW. The sequence data were also analyzed using the MEGALIGN program from LASERGENE (DNASTAR, Madison, WI) in order to evaluate sequence similarities in the huntingtin proteins (Figure 2A).

### HEAT repeat evolution analysis

HEAT repeat consensa were found by searching for the HEAT option with the REP program [41], and loading the individual human, amphioxus and *Ciona intestinalis *huntingtin amino acid sequences. The resulting highly scored consensa were listed, and additional human consensa previously published by Gissi *et al*. [10] were added to the list. By applying the REP program and searching the all consensa option, additional low-score HEAT consensa were found in the three sequences. Therefore, we considered and listed only those corresponding to our multiple alignment (Additional file 2, Tables 1 and 2).

### Whole mount *in situ *hybridisation

To obtain riboprobes for whole mount *in situ *hybridisation, PCR was performed using adult amphioxus cDNA as a template and the HttA_F and HttA_R primers (Figure 1 and Additional file 1, probe A). The resulting 1345-bp fragment was subcloned into the pCR II TOPO vector (Invitrogen, San Diego, CA), and the orientation of the cloned fragments was confirmed by DNA sequencing. Two further riboprobes were prepared using the Htt5 and Htt7 cDNA clones (Figure 1 and Additional file 1, probe B and probe C). Both sense and antisense RNA probes were generated using a digoxigenin (DIG) RNA labelling kit (Roche Diagnostics, Canada) in accordance with the manufacturer's instructions. In order to detect *AmphiHtt *mRNA the probes were used singly and mixed in different experiments. The *in situ *hybridisation experiments were performed at different developmental stages from fertilisation to 48-hour larvae according to Holland *et al*. [42]. Labelled whole mount embryos were photographed using an Olympus IX71 microscope (Olympus Italia s.r.l., Italy), and then counterstained with 1% Ponceau S in 1% acetic acid, dehydrated in ethanol, embedded in Spurr's resin, and serially sectioned at 3–4 μm. Moreover, samples were also examined without Ponceau S counterstaining in order to avoid masking of possible low-level signals. The signal was identical using single or mixed probes, but we only show the results obtained with the mixed probes because the signal was more intense. Negative control experiments were done using sense riboprobes and no specific signal was obtained (Additional file 5).

## Authors' contributions

SC carried out the bioinformatic and molecular analysis and in situ hybridization assays. MT participated to the bioinformatic analyses. SC and MT draft the manuscript. EC and MP provided technical assistance, supervised the research and partecipated in its design. All authors read and approved the final manuscript.

## Supplementary Material

Additional file 1 PCR primers. PCR primers used to isolate a contig of the *AmphiHtt *cDNA sequence. Click here for file

Additional file 2 Huntingtin amino acid alignment. Alignment in fasta format of the huntingtin proteins from chordates. Click here for file

Additional file 3 Rooted tree of huntingtin. Phylogenetic tree created using the neighbor-joining method with *Drosophila melanogaster *huntingtin as the outgroup. Numbers close to the nodes are percentage values represent 1000 bootstrapping. The scale bar of 0.2 at the bottom left corner of the tree indicates 0.2 substitutions for the site. Click here for file

Additional file 4 Exon size of chordate huntingtin genes. Size of protein-coding exons is indicated in basepairs (bp). For amphioxus both scaffolds (scf378 and scf613) are shown. Click here for file

Additional file 5 ISH control. ISH control experiments on amphioxus embryos using sense riboprobes. Click here for file

## References

[B1] Huntington's Disease Collaborative Research Group (1993). A novel gene containing a trinucleotide repeat that is expanded and unstable on Huntington's disease chromosomes. Cell.

[B2] Okazawa H (2003). Polyglutamine diseases: a transcription disorder?. Cell Mol Life Sci.

[B3] Perutz MF, Johnson T, Suzuki M, Finch JT (1994). Glutamine repeats as polar zippers: their possible role in inherited neurodegenerative diseases. Proc Natl Acad Sci USA.

[B4] Steffan JS, Agrawal N, Pallos J, Rockabrand E, Trotman LC, Slepko N, Illes K, Lukacsovich T, Zhu YZ, Cattaneo E, Pandolfi PP, Thompson LM, Marsh JL (2004). SUMO modification of Huntingtin and Huntington's disease pathology. Science.

[B5] Cattaneo E, Zuccato C, Tartari M (2005). Normal huntingtin function: an alternative approach to Huntington's disease. Nat Rev Neurosci.

[B6] Andrade MA, Bork P (1995). HEAT repeats in the Huntington's disease protein. Nat Genet.

[B7] Neuwald AF, Hirano T (2000). HEAT repeats associated with condensins, cohesins, and other complexes involved in chromosome-related functions. Genome Res.

[B8] Goehler H, Lalowski M, Stelzl U, Waelter S, Stroedicke M, Worm U, Droege A, Lindenberg KS, Knoblich M, Haenig C, Herbst M, Scherzinger E, Abraham C, Bauer B, Hasenbank R, Fritzsche A, Ludewig AH, Buessow K, Coleman SH, Gutekunst CA, Landewehrmeyer BG, Lehrach H, Wanker EE (2004). A protein interaction network links GIT1, an enhancer of huntingtin aggregation, to Huntington's disease. Mol Cell.

[B9] Li Z, Karlovich CA, Fish MP, Scott MP, Myers RM (1999). A putative *Drosophila *homolog of the Huntington's disease gene. Hum Mol Genet.

[B10] Gissi C, Pesole G, Cattaneo E, Tartari M (2006). Huntingtin gene evolution in Chordata and its peculiar features in the ascidian *Ciona *genus. BMC Genomics.

[B11] Kauffman JS, Zinovyeva A, Yagi K, Makabe KW, Raff RA (2003). Neural expression of the Huntington's disease gene as a chordate evolutionary novelty. J Exp Zool B Mol Dev Evol.

[B12] Karlovich CA, John RM, Ramirez L, Stainier DYR, Myers RM (1998). Characterization of the Huntington's disease (HD) gene homolog in the zebrafish *Danio rerio*. Gene.

[B13] Smith JE, Bullock TH, Horridge GA (1965). Structure and Function in the Nervous Systems of Invertebrates. Echinodermata.

[B14] Cobb JLS (1970). The significance of the radial nerve cords in Asteroids and Echinoids. Z Zellforsch.

[B15] Cavey MJ, Markel K, Harrison FW, Chia FS (1994). Echinoidea. Microscopic Anatomy of Invertebrates.

[B16] Zuccato C, Tartari M, Crotti A, Goffredo D, Valenza M, Conti L, Cataudella T, Leavitt BR, Hayden MR, Timmusk T, Rigamonti D, Cattaneo E (2003). Huntingtin interacts with REST/NRSF to modulate the transcription of NRSE-controlled neuronal genes. Nat Genet.

[B17] Blair JE, Hedges SB (2005). Molecular phylogeny and divergences times of deuterostome animals. Mol Biol Evol.

[B18] Delsuc F, Brinkmann H, Chourrout D, Philippe H (2006). Tunicates and not cephalochordates are the closest living relatives of vertebrates. Nature.

[B19] Bourlat SJ, Juliusdottir T, Lowe CJ, Freeman R, Aronowicz J, Kirschner M, Lander ES, Thorndyke M, Nakano H, Kohn AB, Heyland A, Moroz LL, Copley RR, Telford MJ (2006). Deuterostome phylogeny reveals monophyletic chordates and the new phylum Xenoturbellida. Nature.

[B20] Holland LZ, Gibson-Brown JJ (2003). The *Ciona *intestinalis genome: when the constraints are off. BioEssays.

[B21] Rockabrand E, Slepko N, Pantalone A, Nukala VN, Kazantsev A, Marsh JL, Sullivan PG, Steffan JS, Sensi SL, Thompson LM (2007). The first 17 amino acids of Huntingtin modulate its sub-cellular localization, aggregation and effects on calcium homeostasis. Hum Mol Genet.

[B22] Tandem repeats Finder program. http://tandem.bu.edu/trf/trf.submit.options.html.

[B23] Lacalli TC, Kelly SJ (1999). Somatic motoneurons in the anterior nerve cord of amphioxus larvae: cell types, cell position and innervation patterns. Acta Zool.

[B24] Bardet PL, Schubert M, Horard B, Holland LZ, Laudet V, Holland ND, Vanacker JM (2005). Expression of estrogen-receptor related receptors in amphioxus and zebrafish: implications for the evolution of posterior brain segmentation at the invertebrate-to-vertebrate transition. Evol Dev.

[B25] Garcia-Fernandez J, Holland PHW (1994). Archetypal organization of the amphioxus *Hox *gene cluster. Nature.

[B26] Minguillon C, Ferrier DE, Cebrian C, Garcia-Fernandez J (2002). Gene duplications in the prototypical cephalochordate amphioxus. Gene.

[B27] Duyao MP, Auerbach AB, Ryan A, Persichetti F, Barnes GT, McNeil SM, Ge P, Vonsattel JP, Gusella JF, Joyner AL, MacDonald ME (1995). Inactivation of the mouse Huntington's disease gene homolog Hdh. Science.

[B28] Nasir J, Floresco SB, O'Kusky JR, Diewert VM, Richman JM, Zeisler J, Borowski A, Marth JD, Phillips AG, Hayden MR (1995). Targeted disruption of the Huntington's disease gene results in embryonic lethality and behavioural and morphological changes in heterozygotes. Cell.

[B29] Zeitlin S, Liu JP, Chapman DL, Papaioannou VE, Efstratiadis A (1995). Increased apoptosis and early embryonic lethality in mice nullizygous for the Huntington's disease gene homolog. Nat Genet.

[B30] Zhang SC, Holland ND, Holland L (1997). Topographic changes in nascent and early mesoderm in amphioxus embryos studied by DiI labeling and by *in situ *hybridization for a *Brachyury *gene. Dev Genes Evol.

[B31] White JK, Auerbach W, Duyao MP, Vonsattel JP, Gusella JF, Joyner AL, MacDonald ME (1997). Huntingtin is required for neurogenesis and is not impaired by the Huntington's disease CAG expansion. Nat Genet.

[B32] *B. floridae *genome assembly (v1.0). http://genome.jgi-psf.org/Brafl1/Brafl1.home.html.

[B33] GenomeScan. http://genes.mit.edu/genomescan.html.

[B34] GENSCAN. http://genes.mit.edu/GENSCAN.html.

[B35] GMAP. http://www.gene.com/share/gmap/.

[B36] Wise2. http://www.ebi.ac.uk/Wise2/.

[B37] Thompson JD, Higgins DG, Gibson TJ (1994). CLUSTAL W: improving the sensitivity of progressive multiple sequence alignment through sequence weighting, position-specific gap penalties and weight matrix choice. Nucleic Acids Res.

[B38] Abascal F, Zardoya R, Posada D (2005). ProtTest: selection of best-fit models of protein evolution. Bioinformatics.

[B39] Guindon S, Gascuel O (2003). A simple, fast, and accurate algorithm to estimate large phylogenies by maximum likelihood. Syst Biol.

[B40] Kumar S, Tamura K, Nei M (2004). MEGA3: Integrated software for Molecular Evolutionary Genetics Analysis and sequence alignment. Briefings in Bioinformatics.

[B41] REP program. http://www.embl-heidelberg.de/~andrade/papers/rep/search.html.

[B42] Holland LZ, Holland PWH, Holland ND, Ferraris JD, Palumbi SR (1996). Revealing homologies between body parts of distantly related animals by in situ hybridization to developmental genes: amphioxus versus vertebrates. Molecular Zoology: Advances, Strategies, and Protocols.

[B43] Lacalli TC, Holland ND, West JE (1994). Landmarks in the anterior central nervous system of amphioxus larvae. Philos Trans R Soc Lond B.

[B44] Lacalli TC (1996). Frontal eye circuitry, rostral sensory pathways and brain organization in amphioxus larvae: Evidence from 3D reconstructions. Philos Trans R Soc Lond B.

[B45] Olsson R, Oksche A, Rodrìguez EM, Fernandez-Llebrez P (1993). Reissner's fiber mechanisms: Some common denominators. The subcommissural organ.

[B46] Jackman WR, Langeland JA, Kimmel CB (2000). Islet reveals segmentation in the amphioxus hindbrain homolog. Dev Biol.

[B47] Jackman WR, Kimmel CB (2002). Coincident iterated gene expression in the amphioxus neural tube. Evol Dev.

