# Supplementary material for: Characterization, developmental expression and evolutionary features of the huntingtin gene in the amphioxus Branchiostoma floridae
Source: BMC Dev Biol. 2007 Nov 15;7:127. doi: 10.1186/1471-213X-7-127 (PMC2206037; doi:10.1186/1471-213X-7-127)
Supplement: Additional file 1 — PCR primers. PCR primers used to isolate a contig of the AmphiHtt cDNA sequence. [file 1471-213X-7-127-S1.pdf]

Table 1

Oligo name	Oligo Sequence (5'-3')	T (°C) Annealing
HttF1	TGA AGG TGT AGC AAT CTA AGA TGG	55
HttR1	TGC AGG ACA AGC TCG AAA ATC TG	55
HttF2	GCT GGC TGC TGA CTG TCT TAT TGG	59
HttR2	GAC AGG GCC TTC ACG CTC ACA CG	59
HttF3	GCG AGG TCC GTG TGA GCG TGA A	59
HttR3	AGG GAG GGG AGG GCA GTC TTA GCA	59
HttF4	CTG GCC AGG GTC GTC AAT GTC T	60
HttR4	CTC CTT CCC CTG GTC AGC CTT ACT	60
HttF5	ATC CCG CCC ATC TTC CAC TTC	58
HttR5	GTT CCA CAT CGC TCA GGT TCT	58
HttF6	CGA GAT CAT GCA GAC CC CCA AGA G	60
HttR6	AGG TCC AGC CAA CAG CAT TTA TCC	60
HttF7	GGG GGC ATC ACC TGG GGG AGA G	63
HttR7	CCT GGG CCG GGA AGA AGT CGT C	63
HttF8	ATG CTG ACC TGT ATG TAC ACC	51
HttR8	TCA GTG GAT GAG TTG GAT GAG	51
HttA_F	GGC CAC CAC GGA AAA ACT GCT	60
HttA_R	CCC CCT CCC CAC CCG TCT GTA	60
