# Supplementary figures and images for: Characterization, developmental expression and evolutionary features of the huntingtin gene in the amphioxus Branchiostoma floridae
Source: BMC Dev Biol. 2007 Nov 15;7:127. doi: 10.1186/1471-213X-7-127 (PMC2206037; doi:10.1186/1471-213X-7-127)

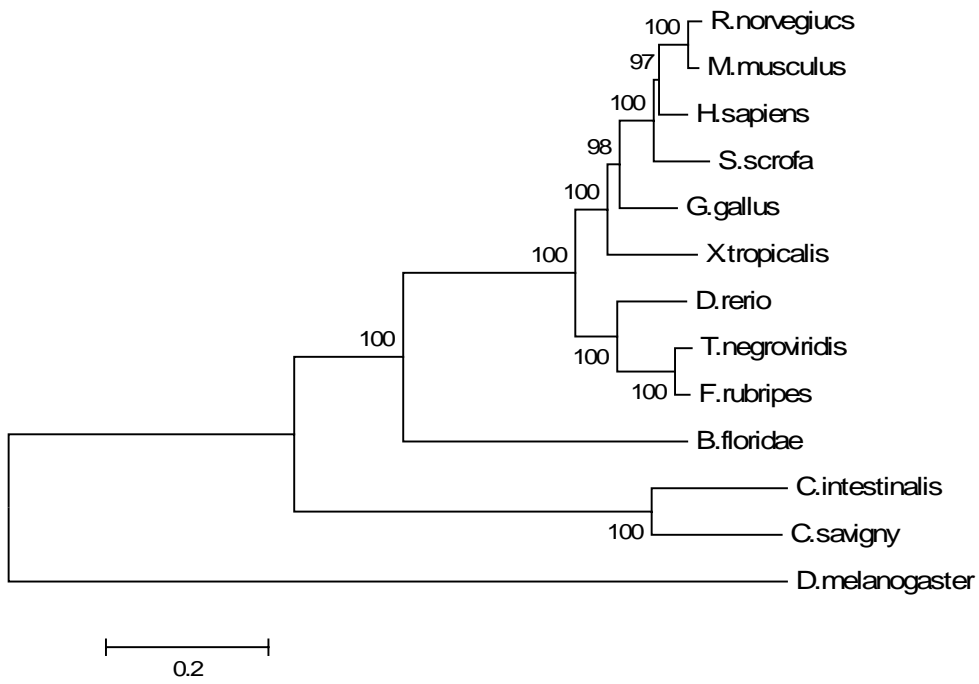

Supplement: Additional file 3 — Rooted tree of huntingtin. Phylogenetic tree created using the neighbor-joining method with Drosophila melanogaster huntingtin as the outgroup. Numbers close to the nodes are percentage values represent 1000 bootstrapping. The scale bar of 0.2 at the bottom left corner of the tree indicates 0.2 substitutions for the site. [file 1471-213X-7-127-S3.pdf]

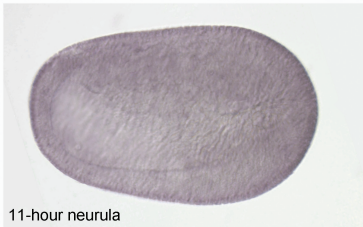

11-hour neurula

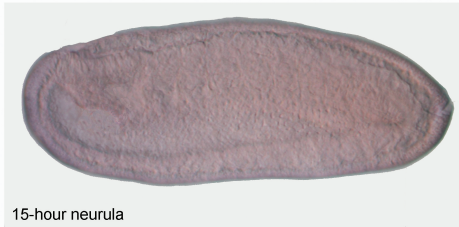

15-hour neurula

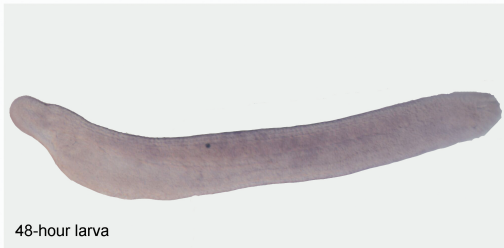

48-hour larva

Supplement: Additional file 5 — ISH control. ISH control experiments on amphioxus embryos using sense riboprobes. [file 1471-213X-7-127-S5.pdf]
